# Combination Therapy using Co-encapsulated Resveratrol and Paclitaxel in Liposomes for Drug Resistance Reversal in Breast Cancer Cells *in vivo*

**DOI:** 10.1038/srep22390

**Published:** 2016-03-07

**Authors:** Jie Meng, Fangqin Guo, Haiyan Xu, Wei Liang, Chen Wang, Xian-Da Yang

**Affiliations:** 1 Key Laboratory for Biological Effects of Nanomaterials and Nanosafety, National Center for Nanoscience and Technology of China, Beijing, China; 2 South China Normal University, Guangdong, China; 3 Institute of Basic Medical Sciences, Chinese Academy of Medical Sciences and Peking Union College, Beijing, China; 4 Institute of Biophysics, Beijing, China

## Abstract

Multidrug resistance (MDR) is a major impediment to cancer treatment. A promising strategy for treating MDR is the joint delivery of combined anticancer agents to tumor cells in a single nanocarrier. Here, for the first time, Resveratrol (Res) was co-encapsulated with paclitaxel (PTX) in a PEGylated liposome to construct a carrier-delivered form of combination therapy for drug-resistant tumors. The composite liposome had an average diameter of 50 nm with encapsulated efficiencies of above 50%. The studies demonstrated that the composite liposome could generate potent cytotoxicity against the drug-resistant MCF-7/Adr tumor cells *in vitro* and enhance the bioavailability and the tumor-retention of the drugs *in vivo*. Moreover, systemic therapy with the composite liposome effectively inhibited drug-resistant tumor in mice (*p* < 0.01), without any notable increase in the toxicity. These results suggested that the co-delivery of Res and a cytotoxic agent in a nanocarrier may potentially improve the treatment of drug-resistant tumors.

Cancer is the single largest cause of death in numerous countries, which annually claims more than 6 million lives worldwide. Multidrug resistance (MDR) is considered a major impediment to cancer treatment because most cancer-related deaths are due to metastatic tumor resistant to chemotherapy^1^. MDR contributes to the failure of chemotherapies for various cancers, including breast, ovarian, lung, gastrointestinal, and hematological malignancies^2^. Multiple mechanisms of tumor cells have been associated with drug resistance, including the increased drug efflux, decreased drug intake, activation of detoxifying systems, activation of DNA repair process, and the evasion of drug-induced apoptosis^3^. Despite numerous attempts to overcome MDR, the treatment of drug-resistant cancer remains a major medical issue^4^,^5^.

An essential strategy for treating drug-resistant tumors is the use of a combination of multiple anticancer agents. The complicated molecular pathways of cancer have interconnected routes with multiple redundancies^6^. Single-drug therapy often triggers and reinforces alternative molecular pathways in cancer cells, thereby leading to drug-resistance mutations and tumor relapse^7^. Combination therapy has long been adopted as the standard first-line treatment of several malignancies to improve the clinical outcome. Combination therapy with anticancer drugs has been shown to generally induce synergistic drug actions and deter the onset of drug resistance^1^,^8^.

The co-delivery of multiple anticancer agents via a nanocarrier is a promising approach to further improve the effectiveness of combination therapy. The effects of standard combination therapy are often limited by the different pharmacokinetics of the drugs, thereby causing the uncoordinated uptake of various drugs by the tumor cell and reducing their synergistic anticancer effects^6^. Moreover, evidence suggests that the synergy of combined drugs is highly dependent on the relative concentrations of drugs, thereby rendering it difficult to optimize the dosage and schedule of conventional combination therapies^9^. The co-delivery of multiple anticancer agents using a nanocarrier may overcome some of these difficulties. Nanocarriers can deliver several drugs to the same tumor cell in one package, thereby promoting their synergistic action against the targeted cancer cell. Nanocarriers may deliver a relatively high drug load, which may overwhelm drug efflux mechanisms of cancer cells, thereby enhancing the anticancer effect of the drug^10^,^11^. Furthermore, drug-loaded nanoparticles may accumulate more at the tumor site, which is secondary to the enhanced permeability and retention (EPR) effect^12^,^13^. Preclinical studies have shown that multidrug-loaded nanocarriers can reverse drug resistance more efficiently than conventional combination therapies^6^. Commonly used nanocarrier platforms include liposomes, polymeric micelles, dendrimers, and mesoporous silica particles^14^. A cytotoxic anticancer drug is usually co-administered with another therapeutic agent to overcome MDR. The most commonly co-administered agents are chemosensitizers that inhibit drug efflux pumps in tumor cells, including cyclosporine A (CyA), verapamil, or tariquidar^15^. Other reported co-administered agents include pro-apoptosis compounds, antiangiogenic agents, small interference RNAs (siRNA), or another cytotoxic agent^6^.

An issue regarding the use of efflux pump modulators such as chemosensitizers is the additional toxicity associated with these agents. The use of CyA may generate adverse effects, such as immunosuppression, leucopenia, nephrotoxicity, or glomerular capillary thrombosis^16^. Verapamil and other calcium-channel blockers may produce adverse effects such as dizziness, fatigue, congestive heart failure, hypotension, or arrhythmia^17^. Preclinical studies have shown that the concentration of calcium channel blockers (such as verapamil) for MDR reversal is toxic to normal cells; these compounds may have hemodynamic side effects^18^. Such potential toxicities limit the use of the above chemosensitizers with standard chemotherapies in advanced cancer patients^19^. Thus, the identification of chemosensitizers that are less toxic, or even beneficial, to the body is medically important for their co-administration with cytotoxic agents in combination therapies against drug-resistant tumors.

Resveratrol (Res) is a natural phytoalexin that has attracted attention because of its potential health benefits^20^,^21^. Res is widely distributed in plants such as grapes, berries, plums, and peanuts. The anticancer, anti-inflammatory, anti-oxidant, anti-aging, blood sugar-lowering, and beneficial cardiovascular effects of Res have been reported in animal models^20^,^22^. Previous *in vitro* and animal studies have showed that Res has antiproliferative and pro-apoptotic activity against several types of cancer^23^, including melanoma^24^, lung cancer, neuroblastoma, and prostate cancer^25^,^26^. In addition, Res has demonstrated its antiproliferative activity against tumor cells with multidrug resistance^27^. However, to the best of our knowledge, the co-delivery of Res with a cytotoxic agent via a nanocarrier has not been described in the literature as an approach to overcome MDR. In this study, Res and paclitaxel (PTX) were co-entrapped in a nanoscale liposome, and evaluated for MDR reversal using *in vitro* and *in vivo* tumor models. Liposomes were chosen as the nanocarriers because they have gained FDA approval and represent a mature technology with proven clinical efficacy. The PEGylated liposome (stealth liposome), in particular, is an efficient drug carrier that can evade rapid clearance by the reticuloendothelial system of the body^28^. Liposomal formulations of doxorubicin (Doxil, Myocet) and daunorubicin (DaunoXome) have been approved for the treatment of metastatic breast cancer and AIDS-related Kaposi’s sarcoma. To date, liposomes are the only nanoparticle-based combinatorial drug delivery platforms that have been used in clinical trials^29^. Here, we report that co-entrapped Res and PTX in a liposome (referred to as a composite liposome) could effectively reverse the drug-resistance of tumor cells in *ex vivo* and *in vivo* studies.

## Materials and Methods

### Preparation and characterization of liposome

#### Liposome preparation

Phosphatidylcholine (PC) was purchased from Merya Company (Beijing, China); DSPE-mPEG2000 was provided by Avanti Polar Lipids (Alabaster, AL, USA). PTX was purchased from Norzer Pharmaceutical Co. Ltd. (Beijing, China). Res was purchased from Hong Jiang Hua Guang Biotech Co. Ltd. (Hunan, China). To prepare the blank liposomes, 640 mg of PC and 90 mg of DSPE-mPEG2000 were dissolved in chloroform. For the drug-carrying liposomes, 640 mg of PC and 90 mg of DSPE-mPEG200 were dissolved in chloroform, with 8 mg of PTX and/or 20 mg of Res. The resulting solution was evaporated to dryness. The dried lipid film was subsequently emulsified in 10 ml saline using a sonicator (for five sonications, each for 15 s at 20 W/cm^2^, with a 5 s pause between each sonication). Titanium particles released by sonication tips were removed by filtration.

#### Morphological study and particle size measurement

The morphology of the composite liposomes was observed by transmission electron microscopy (TEM; H-600; Hitachi, Japan). Freshly prepared liposomes were diluted with distilled water, dropped onto a 200-mesh copper grid, and stained with 1% sodium phosphotungstate solution for 2 min. The samples were incubated at room temperature until a dried film was obtained. The air-dried samples were directly examined by TEM. The particle size distribution of the composite liposome was evaluated using a dynamic light scattering detector (Zetasizer Nano-ZS; Malvern, UK).

#### Evaluation of encapsulation efficiency and *in vitro* drug release profile

After preparing the liposome, the un-encapsulated PTX and Res were removed using a Sephadex G-50 gel-filled column (20 cm × 1.0 cm). The drug-carrying liposomes were dissolved with Triton X-100, and the amount of PTX or Res was determined using a high-performance liquid chromatography (HPLC) system (Agilent Technologies Inc., Cotati, CA, USA). A Waters C18 Symmetry column (180 mm × 4.6 mm) was used as the analytical column. The eluent contained a mixture of methanol and water (70:30, v/v). The flow rate was set at 1.0 mL/min. The amount of PTX and Res were detected at absorption wavelengths of 227 and 306 nm, respectively. The drug encapsulation efficiency (EE%) of PTX or Res was calculated as the percentage ratio of *F*_i_/*F*_t_, where *F*_i_ is the concentration of entrapped PTX or Res, and *F*_t_ is the initial concentration of PTX or Res. The release of PTX or Res from the composite liposome was evaluated by incubating 1 mL of the liposome in a dialysis bag immersed in 20 mL of phosphate-buffered solution (PBS; pH 7.4). The entire system was stirred at 37 °C. The amount of PTX or Res released at each time point (0, 1, 2, 4, 8, 12, 24, 48, and 72 h) was determined by HPLC.

### Experiments with tumor cell lines

#### Cell Culture

The human breast cancer cell line MCF-7 was provided by the Cancer Institute and Cancer Hospital of the Chinese Academy of Medical Sciences. The human multidrug-resistant breast cancer cell line MCF-7/Adr was obtained from the Shanghai Bioleaf Biotech Co., Ltd. All cell lines were cultured in RPMI 1640 (Gibco Inc., USA), supplemented with 10% fetal bovine serum (Gibco Inc., USA) with humidified incubation under 5% CO_2_ at 37 °C.

### *In vitro* anti-proliferation study

The isolated MCF-7 or MCF-7/Adr cells were used to seed the microtiter plates (96 wells, flat-bottomed; Corning) at cell densities of 10^3^ and 10^4^ cells/well, respectively. After 8 h incubation, the cultures were exposed to treatments of saline, the blank liposome, Res liposome, PTX liposome, or the composite liposome. The PTX concentration was set at 1.5 μg/mL; the Res concentration was set at 1, 3, 5, and 10 μg/mL. After 24, 48, and 72 h of incubation, the cell viability was measured using a standard Cell Counting Kit-8 assay (CCK-8; Dojindo Laboratories, Japan), according to manufacturer’s protocol. The optical density (OD) was obtained at 450 nm using a Spectra Max M2 apparatus (Molecular Devices Inc., CA, USA). The cell viability (%) was determined using the following equation:


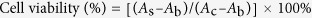


where *A*_s_ is the OD of the test well. *A*_b_ and *A*_c_ are the ODs of the blank liposome and the control (culture medium, CCK-8, without toxicant) wells, respectively. Each independent experiment was repeated for six times.

### Animal Experiments

#### *In vivo* anti-tumor study

BALB/c nude mice (female, weighing 16.0 ± 1.0 g) were purchased from the Vital River Laboratory Animal Technology Co., Ltd. (China) and maintained under specific pathogen-free conditions. The MCF-7 (0.2 mL, 1 × 10^5^ cells/mL) and MCF-7/Adr (0.2 mL, 1 × 10^7^ cells/mL) cell suspensions were injected subcutaneously into the corresponding mice. Tumors were allowed to grow for approximately five days to a volume of 50 mm^3^ to 100 mm^3^, as measured with calipers, before starting the treatments. The tumor-bearing mice were randomly assigned to five experimental groups (with 3 mice per group). The saline, blank liposome, PTX liposome (8 mg/kg), Res liposome (20 mg/kg), and composite liposome (PTX at 8 mg/kg, Res at 20 mg/kg) treatments were then intravenously administered via the tail vein, every two days. The body weight and tumor size were monitored during the treatment course. The mice were sacrificed on day 14; the tumors were immediately harvested, weighed, and photographed.

The mouse experiments were conducted in accordance with the guide for the care and use of medical laboratory animals (Ministry of Health, China) and were approved by the Biomedical Research Ethics Committee of Beijing University.

### Pharmacokinetic research and distribution of drugs-encapsulated liposome *in vivo*

Additional nude mice bearing the MCF-7/Adr tumors (volume, 50 mm^3^ to 100 mm^3^) were used to examine the tissue distribution of the drugs *in vivo*. The treatment groups were administered with free PTX, free Res, the PTX liposome, Res liposome, and the composite liposome. Treatments were administered using the same protocol used in the *in vivo* anti-tumor study. In each treatment group, mice were sacrificed at 24 h, 48 h, and 2 wk after treatment initiation (*n* = 6 at each time point). Organ tissues including those of the tumor, liver, kidney, lung, heart, and spleen were removed and washed twice with physiological solution (0.9% NaCl). These tissues were examined for the concentration of PTX or Res by HPLC. Pharmacokinetic studies were likewise conducted using nude mice. The mice received one intravenous injection of the designated drug, in its proper formulation and at a predetermined dosage of 8 mg/kg of PTX and/or 20 mg/kg of Res. At 1, 3, 5, 12, 24, and 48 h after injection, blood samples were collected from all mice in each group, and the concentration of PTX or Res in each sample was measured by HPLC.

### Statistical analysis

The mean and standard deviation of each dataset were obtained. Post hoc analysis by Dunnett’s *t*-test was used to assess the statistical significance of data. Differences were considered statistically significant at *p* < 0.05. Data analysis was performed using the PASW Statistics (version 18) software (SPSS Inc., IL, USA).

## Results and Discussion

### Characterization of composite liposome

**Figure 1:**
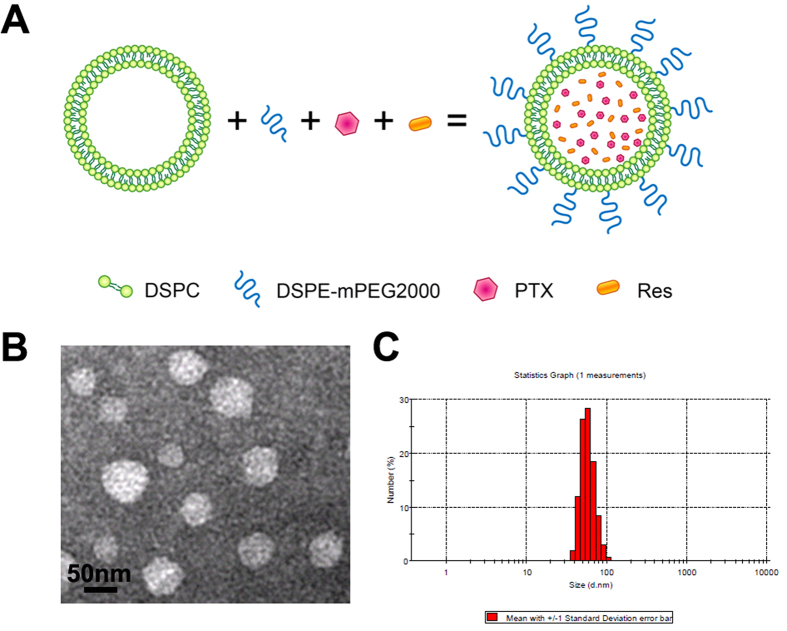
Design and characterization of the composite liposome. (**A**) Scheme and the components of the liposome. (**B**) TEM image of the prepared composite liposome. (**C**) Size distribution spectrum, as determined by the laser diffraction size detector.

The PEGylated liposome containing Res and/or PTX was prepared in this study using the standard ultrasonic dispersion method. One drawback of the unmodified liposome is its rapid clearance from the blood via the reticuloendothelial system of the liver and spleen. Thus, the overall efficacy of the liposome as a drug carrier is limited. Modification of the liposome surface with PEG may reduce the capture of liposome by the liver or spleen, thereby producing a log-circulating liposome (stealth liposome) that can function as an efficient drug carrier with proven clinical efficacy^28^. As illustrated in Fig. 1A, PTX and Res were co-encapsulated in a composite liposome; DSPE-mPEG2000 was incorporated into the outer membrane to improve the stability and stealth of the liposome.

The size of the drug-loaded liposome is an important feature that determines whether a drug carrier will be sequestrated by the reticuloendothelial system; nanocarriers larger than 200 nm are predisposed to capture by macrophages in the liver and spleen^29^. The size of the composite liposome was evaluated using TEM and DLS in this study. As shown in Fig. 1B, TEM revealed that the liposomes were moderately uniform and spherical in shape (Fig. 1B). The average liposome size as observed by TEM was approximately 50 nm. The distribution of the liposome size was measured by the DLS method (Fig. 1C), which again yielded an average liposome size of approximately 50 nm (range, 25 nm to 102 nm). This size presumably favors the reduced capture of liposome by macrophages in the liver and spleen.

### Encapsulation efficiency and release rate of composite liposome

To determine the amount of PTX or Res encapsulated by a liposome, the encapsulation efficiencies (EE) of liposomes that contain Res and/or PTX were measured. The unentrapped PTX or Res was separated from the liposome suspension by a sepharose column. The drug contents of the liposomes were analyzed by HPLC. As presented in Table 1, single-component liposomes exhibited efficient encapsulating ability for either PTX or Res, with EE reaching levels of 90.3% ± 2.4% and 92% ± 3.0%, respectively. For composite liposomes with both PTX and Res, the EE was 52% ± 3.7% for PTX and 56% ± 3.3% for Res. These results indicated that PTX and Res could be loaded together into a composite liposome, thereby achieving an EE greater than 50%. Thus, the composite liposome is a feasible method for the co-delivery of both agents to tumor cells in one package.

**Figure 2:**
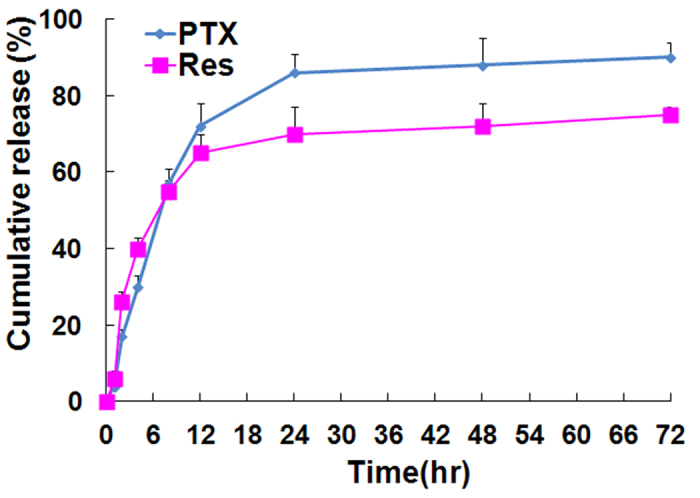
Cumulative release of PTX or Res from the composite liposome at 37 °C in PBS (pH 7.4; *n* = 3, mean ± standard deviation).

To evaluate whether PTX and/or Res could be released by the composite liposome, the standard drug release rate (RR) was measured for each agent at 37 °C in PBS (pH 7.4). The cumulative release curves of Res and PTX from the composite liposome are presented in Fig. 2. The two components exhibited similar release patterns. Relatively faster release of PTX and Res was observed from the composite liposome in the first 24 h (PTX, 86.4% ± 3.6%; Res, 71.8% ± 4.5%). At 48 h, the cumulative release reached 89.3% ± 2.9% for PTX, and 76.7% ± 4.9% for Res. The composite liposome was shown to have a sustained release profile that was consistent with most liposomal drug carriers, as described in previous studies^30^.

### Cytotoxicity studies *in vitro*

**Figure 3:**
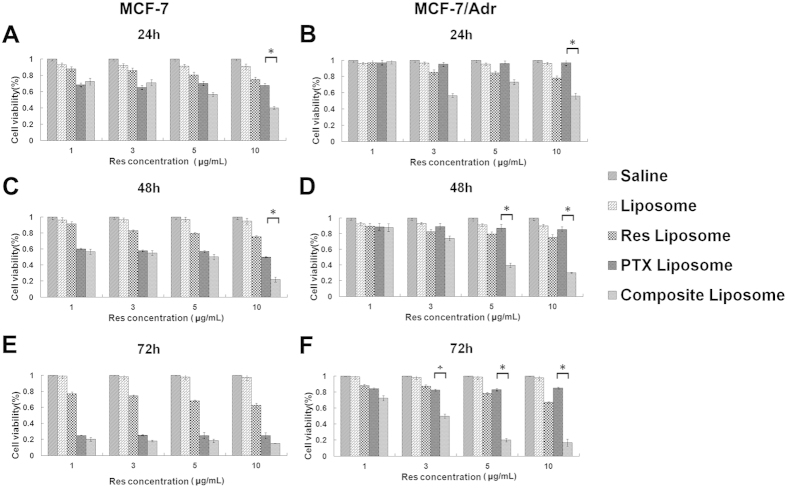
*In vitro* cytotoxicity on MCF-7 (**A,C,E**) and MCF-7/Adr (**B,D,F**) cells of the saline solution, liposome, Res liposome, PTX liposome, and the composite liposome at 24 h (**A,B**), 48 h (**C,D**), and 72 h (**E,F**). The PTX concentration was fixed at 1.5 μg/mL, whereas the Res concentrations varied at 1, 3, 5, and 10 μg/mL. Data represent mean ± standard deviation (*n* = 6).

To evaluate the *in vitro* antitumor efficacy of liposome-encapsulated drugs against drug-sensitive and drug-resistant cancer cells, standard viability assay was utilized. The cytotoxic effects of treatment with the blank liposome, Res liposome, PTX liposome, or the composite liposome, with the saline solution as the control, were measured for MCF-7 and MCF-7/Adr cells. Res exhibits poor water solubility, and free Res or PTX were found to be less efficient in producing cytotoxicity than liposomal formulations (data not shown), Thus, only the liposomal drugs were included in these groups. As shown in Fig. 3, the MCF-7 and MCF-7/Adr cells were exposed to liposome-containing PTX (1.5 μg/mL) and/or Res of various concentrations (1, 3, 5, and 10 μg/mL) for 24, 48 and 72 h. The control treatment with the saline solution or the blank liposome had no significant effects on the cell viability of both lines (Fig. 3A to 3F).

For drug-sensitive MCF-7 cells (Fig. 3A,C,E), the Res liposome exhibited mild cytotoxicity, whereas the PTX liposome and composite liposome clearly demonstrated cytotoxicity. Tumor inhibition was time-dependent and generally more prominent at 48 and 72 h. Cancer cell inhibition was likewise found to be dose-dependent, such that the cytotoxicity of Res tended to increase in higher concentrations. Thus, the composite liposome containing PTX and a higher dose of Res exhibited the greatest cytotoxicity.

However, liposomal PTX failed to induce a significant tumor inhibition in drug-resistant MCF-7/Adr cells (Fig. 3B,D,F). Furthermore, liposomal Res produced minimal inhibition. Only the composite liposome containing PTX and Res at the appropriate concentrations could generate significant cytotoxicity. PTX or Res alone was shown to be insufficient for MDR reversal. The synergistic action of both agents was required to eliminate the drug-resistant cancer cells. Interestingly, the composite liposome containing PTX and Res was the most effective formulation against the drug-sensitive and drug-resistant cancer cells, thereby indicating that the combination therapy potentially possessed a broad range of applications in cancer treatment.

### Pharmacokinetic profile and tissue distribution

**Figure 4:**
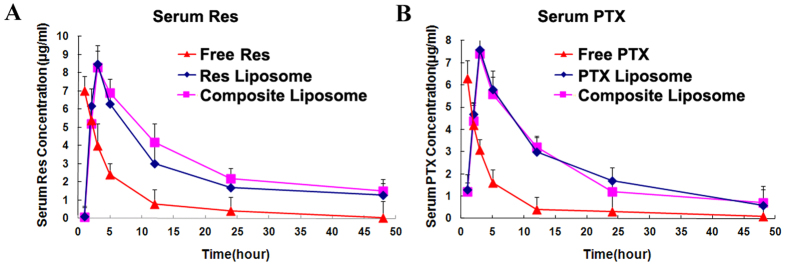
Serum concentration–time profiles of Res (**A**) and PTX (**B**) in nude mice, after intravenous injection of free and liposomal PTX or Res, as well as the composite liposome with both PTX and Res.

To evaluate the pharmacokinetics of the liposomal formulations, mice with MCF-7/Adr tumors were injected intravenously with the free PTX (8 mg/kg), free Res (20 mg/kg), PTX liposome (8 mg/kg), Res liposome (20 mg/kg), or the composite liposome (PTX, 8 mg/kg; Res, 20 mg/kg). Blood was collected at 1, 3, 5, 12, 24, and 48 h after injection, and the serum PTX and Res levels were measured by HPLC. As presented in Fig. 4, free PTX or Res was rapidly cleared from the systemic circulation after drug administration. By contrast, liposomal formulations produced higher blood concentrations of PTX and Res, with longer circulation times. The bioavailability was evaluated by calculating the area under the blood concentration curve from 0 h to 48 h (AUC_0–48_). The AUC_0–48_ values of Res and PTX in liposome was 10.8-fold (Fig. 4A; *p* < 0.01) and 12-fold (Fig. 4B; *p* < 0.01) higher than that of free Res and PTX, respectively. The composite liposome generated an AUC_0–48_ value that was similar to that of the single-drug liposomes. Thus, liposomal formulations of Res or PTX had a sustained-release profile *in vivo*, which greatly improved the bioavailability of both agents.

**Figure 5:**
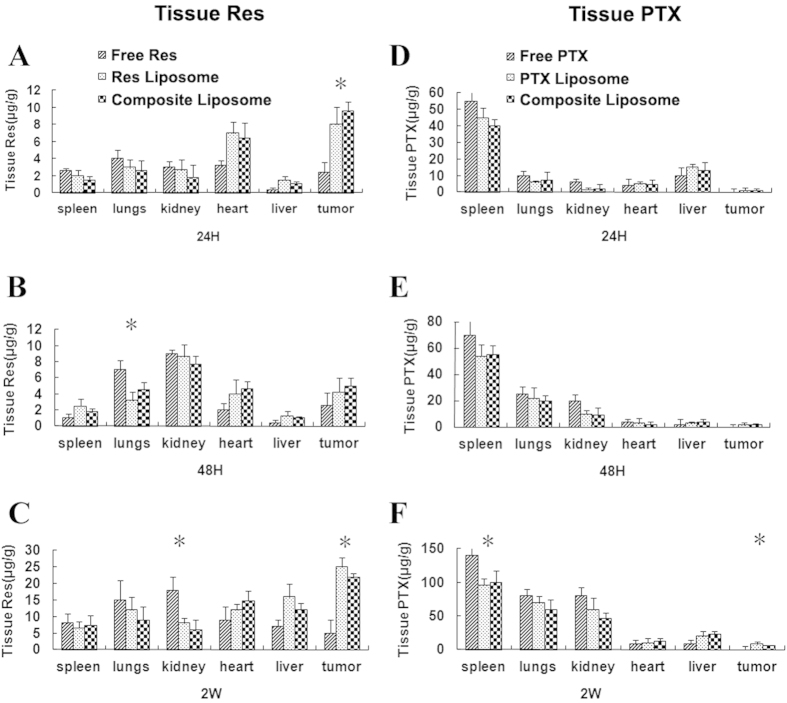
*In vivo* distribution and accumulation of Res (**A** to **C**) and PTX (**D** to **F**) in various organ systems of mice at 24 h (**A,D**), 48 h (**B,E**), and 2 wk (**C,F**) after injection of free and liposomal PTX or Rex, as well as the composite liposome with both PTX and Res.

To further investigate the *in vivo* effects of the liposomal formulations, the biodistribution of PTX and Res in different organ systems were evaluated. Tissue samples from various organs were obtained at 24 h, 48 h, and 2 wk after the regular systemic administration every 48 h of the free or liposome-encapsulated drugs. The Res and PTX contents of these tissues were evaluated by HPLC, and the results are presented in Fig. 5. The Res concentration in the tumor from the composite liposome or Res liposome treatment was 1.6-fold to 5-fold that of the free Res (*p* < 0.05; Fig. 5A to 5C). Notably, the overall amount of accumulated Res was substantially higher in the tumor, as compared with those in other organs, whereas the PTX concentration in the tumor was low, as compared with those in other organs. Therefore, liposome encapsulation may promote the combined action of the two anticancer agents.

### *In vivo* anti-tumor studies

**Figure 6:**
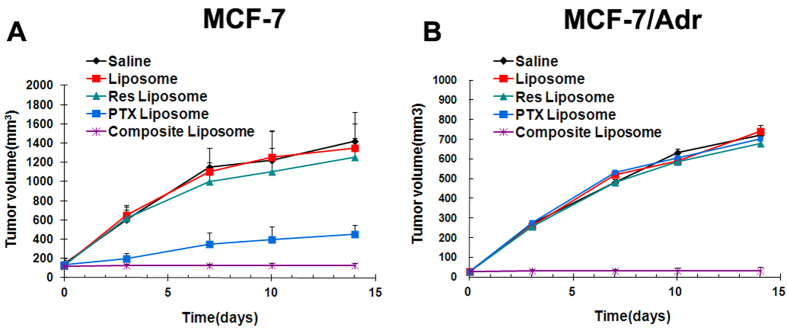
*In vivo* antitumor effects generated by the saline solution, blank liposome, Res liposome, PTX liposome, or the composite liposome with both Res and PTX. Tumor growth curves of MCF-7 (**A**) and MCF-7/Adr (**B**); error bars correspond to 95% confidence intervals. Tumor volumes were measured at 0, 3, 8, 10, and 14 d after tumor cell injection.

To investigate the effectiveness of the combined Res and PTX in the liposome against cancer cells *in vivo*, nude mice with the drug-sensitive MCF-7 or the drug-resistant MCF-7/Adr xenografts were treated using different regimens, including the saline solution (as control), blank liposome, Res liposome, PTX liposome, and the composite liposome containing both Res and PTX. The systemic chemotherapy was administered every 48 h at a dosage of 20 mg/kg Res and/or 8 mg/kg PTX. The tumor volume was measured during the course of treatment, and the results are presented in Fig. 6. For drug-sensitive MCF-7 tumors, the strongest tumor inhibition was demonstrated by the composite liposome. PTX liposome likewise exhibited evident tumor inhibition, whereas Res liposome produced moderate tumor inhibition. The average inhibition ratio was 91%, 77.82%, and 25.81% for the composite liposome, PTX liposome, and the Res liposome, respectively (Fig. 6A). The results suggested that the composite liposome probably induces a synergistic action that leads to more potent tumor inhibition. For the drug-resistant MCF-7/Adr tumors, liposomal PTX or Res failed to clearly demonstrate tumor inhibition. By contrast, the composite liposome with both Res and PTX markedly inhibited tumor growth, with an average inhibition ratio of 81.81% (Fig. 6B). Therefore, the composite liposome has the potential to effectively reverse drug-resistance *in vivo*. A combination of Res and PTX is necessary to achieve the desired therapeutic efficiency against drug-resistant tumors.

**Figure 7:**
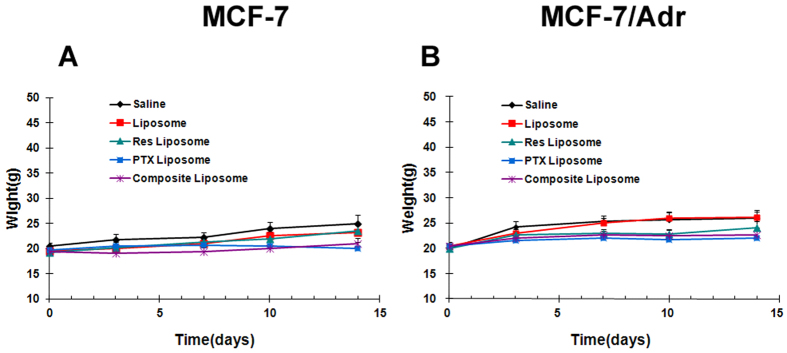
Average body weights of mice bearing MCF-7 (**A**) or MCF-7/Adr (**B**) tumors during the course of therapy. Animals in the various experiment groups received treatments of the saline solution, blank liposome, Res liposome, PTX liposome, and the composite liposome with Res and PTX. Error bars corresponded to 95% confidence intervals.

To compare the adverse effects associated with the various treatments, we recorded the animal body weight during chemotherapy, which is commonly used to evaluate adverse effects. The body weights of mice with either the MCF-7 or MCF-7/Adr tumors were measured at 0, 3, 8, 10, and 14 d after tumor cell injection. As illustrated in Fig. 7, no significant differences were observed in the average body weights of mice treated with the PTX liposome and the composite liposome. Therefore, the toxicity profile of the composite liposome with Res and PTX was comparable with that containing PTX alone.

## Discussion

MDR is a major obstacle to cancer treatment because most cancer-related deaths are attributed to metastases that are resistant to chemotherapy^1^. A primary strategy for treating drug-resistant tumors is a combination of anticancer drugs, but the effectiveness of such combination therapy is often unsatisfactory because of the different pharmacokinetics of the combined drugs^9^. A promising approach for MDR reversal is the use of a nanocarrier that may jointly deliver a combination of drugs to cancer cells, thereby improving the synergy between these therapeutic agents. For this purpose, a cytotoxic anticancer drug is often combined with a chemosensitizer that can promote the effectiveness of the cytotoxic agent^15^. The selected chemosensitizer should not generate significant additional toxicities; the extra adverse effects could limit the applicability of combination therapy as a routine treatment for patients with advanced cancer. Treatment with Res may generate potential health benefits, with only limited toxicity^20^. To our knowledge, this study is the first attempt to co-encapsulate Res with PTX in a PEGylated liposome (Fig. 1A) to build a carrier-delivered combination therapy for the reversal of cancer drug-resistance. The average size of the composite liposome was approximately 50 nm in diameter (Fig. 1B,C). The encapsulation efficiencies of PTX and Res were 52% and 56%, respectively (Table 1). The composite liposome released the drugs in a sustained pattern (Fig. 2). The *in vitro* studies demonstrated that the composite liposome could exhibit potent cytotoxicity against the drug-resistant MCF-7/Adr tumor cells (Fig. 3). The *in vivo* studies showed that the composite liposome improved the bioavailability of the drugs (Fig. 4) and enhanced drug retention in the tumor (Fig. 5). Moreover, the composite liposome with Res and PTX effectively inhibited drug-sensitive and drug-resistant tumors *in vivo* (Fig. 6), without a significant increase in the toxicity (Fig. 7). Therefore, the co-delivery of Res and a cytotoxic agent via a nanocarrier may have potential applications in the treatment of drug-resistant tumors.

The compound Res has been shown to possess antitumor effects; it can influence the three phases of carcinogenesis (initiation, promotion, and progression) by inducing apoptosis, cell cycle arrest, and the suppression of certain transcription factors in cancer cells^23^,^24^. Its antitumor efficacy is exhibited in drug-resistant A549/cDDP cells by inducing apoptosis via mitochondria-dependent signaling pathways^26^. In addition, Res decreases the MRP1 expression in a doxorubicin-resistant leukemia cell line *in vitro*^31^. However, the use of Res for MDR-reversal combination therapy has not been explored thus far, particularly the use of Res with a cytotoxic agent for joint delivery via a nanocarrier. In the present study, Res and PTX were co-encapsulated in a liposome and used to treat drug-resistant tumors. In addition to improving the *in vivo* bioavailability of the drugs, the main advantage of co-encapsulating Res and PTX in the same nanocarrier is the facilitated synergistic effects of the two anticancer drugs. As shown in Fig. 6, neither the liposomal PTX nor the liposomal Res alone could cause significant inhibition of the drug-resistant MCF-7/Adr tumor *in vivo.* By contrast, the composite liposome with both PTX and Res could overcome MDR *in vitro* (Fig. 3B,D,F) and *in vivo* (Fig. 6B). The results suggest that the synergistic action of Res and PTX could lead to the reversal of drug-resistance in the cancer cells. Notably, the composite liposome also demonstrated its improved anticancer effects on the drug-sensitive tumor MCF-7, as compared with the PTX liposome (Fig. 6A). The composite liposome could improve the treatment of drug-resistant and drug-sensitive tumors, thereby suggesting that the co-delivery of Res with PTX generates a synergistic anticancer effect on the tumor, regardless of its MDR status.

As an effective component in the combination therapy, Res may generate less adverse effects than other extensively studied chemosensitizers, notably the drug efflux modulators. The P-glycoprotein (P-gp) modulator CyA is considered one of the most effective MDR-reversing agents^15^, but CyA may cause a wide range of side effects, including gingival hyperplasia, peptic ulcers, hypercholesterolemia, convulsions, immunosuppression, and nephrotoxicity^16^. Verapamil^17^,^32^ and other calcium channel blockers are another class of commonly used chemosensitizers, which can overcome MDR by enhancing the intracellular accumulation of anticancer drugs^33^. Despite the effectiveness of these chemosensitizers for MDR reversal, the potential adverse effects associated with these drugs may limit their routine clinical use in patients with advanced cancer. The high dose of calcium channel blockers required for MDR reversal is usually toxic to normal cells and may cause hemodynamic adverse effects^18^. Thus, novel combination therapies that allow MDR reversal with limited additional toxicity are urgently needed to improve the chance of prospective clinical applications.

As a natural substance produced by plants, Res has attracted much interest because of its potential health benefits. In animal studies, Res has been reported to exhibit anti-inflammatory, anti-oxidant, anti-aging, anticancer, blood sugar-lowering, and cardioprotective effects^22^,^23^,^24^,^25^,^26^,^27^. By jointly delivering Res with a cytotoxic agent via a nanocarrier, the toxicity associated with some chemosensitizers may be reduced. In this study, the liposome containing Res and PTX was shown to exhibit superior MDR reversal (Fig. 6) without additionally generating significant side effects (Fig. 7). These results are in agreement with prior animal studies demonstrating that Res generates health benefits with limited adverse effects^20^.

To improve the clinical feasibility of the proposed combination therapy, a nanocarrier with a reliable track record for efficacy and safety should be chosen. In this study, liposomes were chosen as the carriers for the Res and PTX co-delivery because of its FDA approval and proven clinical efficacy. Liposomal formulations of doxorubicin (Doxil, Myocet) and daunorubicin (DaunoXome) have been approved for the treatment of metastatic breast cancer and AIDS-related Kaposi’s sarcoma. Moreover, liposomes are the only nanoparticle-based combinatorial drug delivery platforms that have entered clinical trials^29^. All the components of the composite liposome in this study have documented human use. PC, DSPE-mPEG2000, and PTX are pharmaceutical components that have been approved for clinical application^34^. Res has likewise been approved for several early-stage clinical trials^35^. In addition, Res-containing products have been sold for years as over-the-counter nutritional supplements for human use. These components were utilized in the present study to develop a carrier-based combination therapy, such that its feasibility for clinical use could be maximized.

Liposome delivery of Res and PTX improved the pharmacokinetics and bioavailability of the two drugs (Fig. 4). Both PTX and Res have poor solubility in water. Although Res is sold as an oral nutritional supplement, it is usually inadequately absorbed and has poor bioavailability when administered orally^36^. The liposome encapsulation of Res and PTX resolved the issue of its poor solubility in water and generated a sustained drug release profile (Fig. 2) that markedly enhanced the *in vivo* bioavailability of the two agents (Fig. 4). Both Res and PTX can be slowly released from the composite liposome, which ensures that the two drugs could work in a coordinated manner to induce MDR reversal and anticancer cytotoxicity.

The liposome size is another important factor that determines successful drug delivery to tumor cells. Nanoscale drug carriers that are larger than 20 nm can generally prevent rapid leakage into the urine, whereas those smaller than 200 nm can evade macrophage capture in the reticuloendothelial system (liver and spleen)^37^. In addition, the size of a gap junction between endothelial cells of the leaky tumor vasculature varies from 100 nm to 600 nm; by virtue of the EPR effect, this size could allow drug-loaded nanoparticles to accumulate more at the tumor site^38^. The composite liposome synthesized in this study had an average size of approximately 50 nm, as measured by TEM and DLS (Fig. 1). This liposome size is presumably advantageous for tumor treatment, because the nanocarrier may evade capture in the liver or spleen but still reach the tumor via the EPR effect. Another factor influencing the *in vivo* biodistribution of a liposome is surface modification. A major drawback of the unmodified liposome is its rapid clearance from the blood by the liver and spleen, thereby limiting its overall value as a drug carrier. Modification of the liposome surface with PEG may retard the capture of liposome by the liver or spleen, thereby prolonging the biological half-life of the PEGylated liposome while improving its accumulation in solid tumors via the EPR effect^12^,^13^. Therefore, a PEGylated composite liposome was constructed and employed in this study. To assess the systemic effects of the drug-loaded liposome, the tissue distribution of Res and PTX were evaluated in this study. The liposome was shown to enhance the accumulation of both Res and PTX in tumor cells by several degrees (Fig. 5). The increased drug accumulation at the tumor site could be attributed to the PEGylated long-circulating liposome and its increasing bioavailability or to the EPR effect of increasing the tumor-retention of the drugs. Interestingly, the use of liposomes increased the amount of PTX in the spleen, but not in the liver. The cause of this discrepancy is unclear and needs further investigation. The PEGylated liposome may have reduced, but not completely avoided, the capture of liposomes by the macrophages in the liver and spleen.

The mechanism for drug-resistance reversal using the liposomal combination of Res and PTX is not entirely clear and may involve several aspects. First, the joint treatment of Res and PTX were necessary for effective action against drug-resistant tumor. Neither the Res nor PTX liposome alone could produce significant inhibition, whereas liposomal combination of Res and PTX resulted in the evident anticancer efficacy and MDR reversal (Fig. 6). Thus, although Res itself was not sufficiently therapeutic, the extra benefits contributed by Res were probably important for facilitating the anticancer effect of PTX and generating MDR reversal. However, the detailed contribution of Res is unclear at this stage. Extensive future studies may be required to elucidate its function. Second, liposome delivery of the anticancer agents increased the drug bioavailability and drug accumulation in tumor tissue. As shown in Fig. 5, liposomal formulations markedly increased the amounts of Res and PTX in the tumor. This phenomenon could be a secondary result of the sustained drug release profile and/or the EPR effect generated by the liposome^12^. Third, the joint delivery of Res and PTX by the same nanocarrier may also contribute to the synergistic action. Different drugs in the appropriate ratios can be carried in one package to tumor cells to satisfy the stringent dose requirements for optimal drug synergy. All these factors may have contributed to the therapeutic efficacy and MDR reversal produced by the carrier-based combination treatment described in this study. Extensive future studies are warranted to unveil the potentially complicated therapeutic mechanisms of Res and PTX co-delivery via composite liposomes.

## Conclusions

MDR is a major obstacle to the treatment of late-stage cancer. The co-delivery of a combination of multiple anticancer drugs by a nanocarrier is a promising therapeutic strategy against drug resistant tumors. In this study, Res and PTX were co-encapsulated in a liposome that was constructed with components approved for human use. The composite liposome reversed the PTX-resistance of MCF-7/Adr tumors and improved the efficacy of both drugs against drug-sensitive (MCF-7) and drug-resistant (MCF-7/Adr) tumors *in vivo*. The results suggest that the co-delivery of Res and cytotoxic agents by a nanocarrier may have potential applications for treating drug-resistant malignancies.

## Additional Information

**How to cite this article**: Meng, J. *et al*. Combination Therapy using Co-encapsulated Resveratrol and Paclitaxel in Liposomes for Drug Resistance Reversal in Breast Cancer Cells *in vivo*. *Sci. Rep.* **6**, 22390; doi: 10.1038/srep22390 (2016).

## Figures and Tables

**Table 1:** Encapsulation efficiency of various liposomes for PTX or Res (*n* = 6).

Agent	PTX liposome	Res liposome	Compositeliposome
PTX	90.3% ± 2.4%	NA	52% ± 3.7%
Res	NA	92% ± 3.0%	56% ± 3.3%

^*^NA: not applicable.
